# The standard diagnosis, treatment, and follow-up of gastrointestinal stromal tumors based on guidelines

**DOI:** 10.1007/s10120-015-0526-8

**Published:** 2015-08-15

**Authors:** Toshirou Nishida, Jean-Yves Blay, Seiichi Hirota, Yuko Kitagawa, Yoon-Koo Kang

**Affiliations:** 1 Department of Surgery, National Cancer Center Hospital East, 6-5-1 Kashiwanoha, Kashiwa, Chiba 277-8577 Japan; 2 Department of Medical Oncology, Centre Leon-Bernard, University Claude Bernard Lyon I, Lyon, France; 3 Department of Surgical Pathology, Hyogo College of Medicine, Nishinomiya, Japan; 4 Department of Surgery, Keio University School of Medicine, Tokyo, Japan; 5 Department of Oncology, Asan Medical Center, University of Ulsan College of Medicine, Seoul, South Korea

**Keywords:** Gastrointestinal stromal tumor, Guidelines, Evidence-based, Consensus based

## Abstract

Although gastrointestinal stromal tumors (GISTs) are a rare type of cancer, they are the commonest sarcoma in the gastrointestinal tract. Molecularly targeted therapy, such as imatinib therapy, has revolutionized the treatment of advanced GIST and facilitates scientific research on GIST. Nevertheless, surgery remains a mainstay of treatment to obtain a permanent cure for GIST even in the era of targeted therapy. Many GIST guidelines have been published to guide the diagnosis and treatment of the disease. We review current versions of GIST guidelines published by the National Comprehensive Cancer Network, by the European Society for Medical Oncology, and in Japan. All clinical practice guidelines for GIST include recommendations based on evidence as well as on expert consensus. Most of the content is very similar, as represented by the following examples: GIST is a heterogeneous disease that may have mutations in *KIT*, *PDGFRA*, *HRAS*, *NRAS*, *BRAF*, *NF1*, or the succinate dehydrogenase complex, and these subsets of tumors have several distinctive features. Although there are some minor differences among the guidelines—for example, in the dose of imatinib recommended for exon 9-mutated GIST or the efficacy of antigen retrieval via immunohistochemistry—their common objectives regarding diagnosis and treatment are not only to improve the diagnosis of GIST and the prognosis of patients but also to control medical costs. This review describes the current standard diagnosis, treatment, and follow-up of GISTs based on the recommendations of several guidelines and expert consensus.

## Introduction

Gastrointestinal stromal tumors (GISTs) are considered potentially malignant tumors and are the commonest mesenchymal tumors in the gastrointestinal tract. Since the discovery of gain-of-function mutations in the *KIT* and *PDGFRA* genes and clinical application of tyrosine kinase inhibitors (TKIs), such as imatinib, our understanding of the molecular and clinical features of GISTs has increased substantially, and the diagnosis and treatment of GIST have rapidly and dramatically changed [1, 2]. These advances provided information that facilitated the preparation of clinical practice guidelines by the National Comprehensive Cancer Network (NCCN) [3] and the European Society for Medical Oncology (ESMO) [4]. Since the first guidelines were published, they have been updated annually or biannually, and other countries have published their own GIST guidelines [5–7]. It is suggested that diagnosis and treatment based on the guidelines will improve the prognosis of patients and the quality of medical care, as well as control medical costs. Last year, the Japanese and ESMO guidelines were updated, and there were consensus meetings of experts in several East Asian countries, including Japan, Korea, Taiwan, and China. This review discusses the current standard diagnosis, treatment, and follow-up of GISTs based on the guidelines and expert consensus [3–7].

## Epidemiology and incidence

The worldwide incidence and prevalence of GIST are estimated to be approximately 1–1.5 per 100,000 per year and 13 per 100,000, respectively [8]. A recent report suggested that, except for incidental GIST, the age-adjusted incidence of clinical GIST was 0.8 per 100,000 per year on the basis of the data from the Surveillance, Epidemiology, and End Results program of the National Cancer Institute [9]. Population-based studies have shown that the median age at diagnosis is in the 60s, although GIST has been detected in all age groups. There is no significant sex difference. GIST in children and young adults, although rare, is a distinct subset of pediatric GIST, and syndromic GISTs may be found in children and individuals in early middle age [10, 11]. The predominant localization of GISTs seems to be the stomach (60 %) and small intestine (30–20 %), but GISTs may develop in the colorectum, esophagus, and, rarely, in the mesentery, omentum, or retroperitoneum (extragastrointestinal GIST), where KIT-positive mesenchymal cells are found.

## Diagnosis

### Clinical presentation

Many GISTs may be identified clinically because of symptoms including gastrointestinal bleeding and subsequent anemia, early satiety, abdominal distension, and discomfort and/or pain due to tumor compression [11]. However, GISTs are sometimes asymptomatic until advanced stages because of a submucosal localization and noninvasive behavior compared with carcinomas. Gastrointestinal examinations, including endoscopy, sometimes reveal asymptomatic GISTs, especially in the stomach. Hence, cancer-screening health examinations may increase the detection of asymptomatic GIST in the stomach [12]. GIST rarely metastasizes to lymph nodes, except for a special subtype of *SDH*-mutated GIST [10], and its spread to the extra-abdominal organs is extremely rare as an initial metastatic presentation.

The natural history of GIST remains largely unknown. Pathology reports on subclinical GISTs have shown an unexpectedly high incidence of microscopic GISTs in the stomach and small intestine [13, 14]. Small GISTs (from a few millimeters to less than 10 mm in diameter) are also commonly found in the proximal stomach of individuals older than 50 years. Immunohistochemistry reveals that these mini-GISTs are KIT-positive, and they often have an oncogenic mutation in the *KIT* or *PDGFRA* gene [15]. Most mini-GISTs are thought to be biologically indolent and do not progress during follow-up unless they have high-risk features such as an irregular border, internal heterogeneity, or ulceration [3, 16]. Although complete surgical resection is the mainstay of treatment for clinical and/or symptomatic GISTs, the clinical significance of surgical treatment remains unknown for asymptomatic and incidentally found mini-GISTs.

### Pathological diagnosis, including rare GISTs

**Fig. 1:**
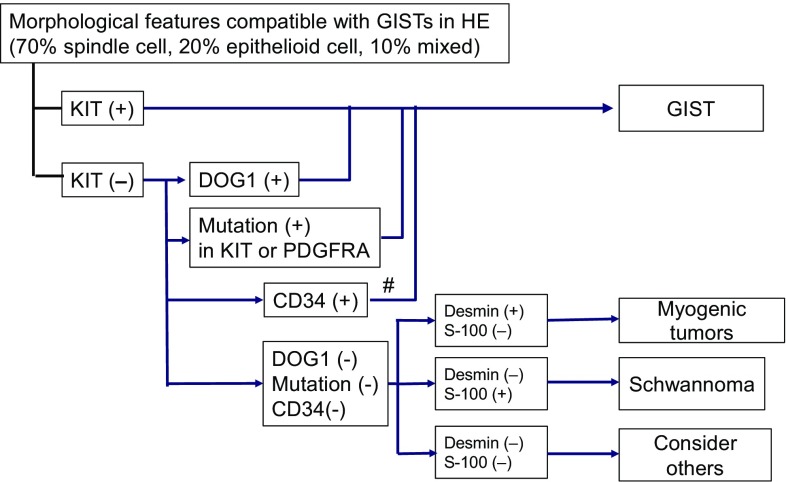
Pathological diagnosis of gastrointestinal stromal tumor (*GIST*) by immunohistochemistry and genotyping. The algorithm for the pathological diagnosis of GIST is shown. The *number sign* means solitary fibrous tumors should be ruled out. *DOG1* discovered on GIST-1, *HE* hematoxylin–eosin staining

The pathological diagnosis of GIST depends on the morphology and immunohistochemical findings. The morphological features include a predominantly spindle cell type (70 %), epithelioid cell type (20 %), or mixed type (10 %). In addition, 95 % of GISTs are positive for KIT (CD117) and/or discovered on GIST-1 (DOG1), and 70 % are found to be positive for CD34 by immunohistochemistry. KIT positivity is a major defining feature for the diagnosis of GIST for a tumor that has morphological features compatible with GIST, although KIT positivity alone is not sufficient for the diagnosis (Fig. 1). When there is KIT negativity, as in approximately 5 % of GISTs, DOG1 staining, followed by CD34 staining, is considered diagnostic. The other important molecular marker that is useful in the diagnosis of GISTs is the presence of mutations in either *KIT* or *PDGFRA*; nearly 80 % and 10 % of GISTs, respectively, are positive for these mutations. When gastric GISTs have no mutations in *KIT* or *PDGFRA*, immunostaining for succinate dehydrogenase (SDH) iron–sulfur subunit (subunit B) (SDHB) is recommended [10]. The mitotic count is of prognostic value and should be expressed as the number of mitoses for a total area of 5 mm^2^, which should replace the conventional 50 high-power-field area. Standardized antigen retrieval is recommended in Japan but not in the NCCN and ESMO guidelines. Evidence of antigen retrieval during KIT immunostaining is lacking. Because GIST is a rare disease and diagnostic concordance among pathologists is not obtainable in some cases [17], consultation with or a second pathological examination by pathologists specializing in sarcoma is recommended in the ESMO guidelines.

### Genotyping

Mutation testing, at least for the *KIT* and *PDGFRA* genes, is recommended when TKIs, such as imatinib, sunitinib, and regorafenib, are to be used. *KIT* mutations (present in 80 % of primary GISTs) are commonest in exon 11 (65 %), followed by exon 9 (8 %), and are rarely found in exons 13 and 17. Most GISTs caused by *KIT* exon 11 or 13 mutations are naïve to imatinib. *KIT* exon 9 mutations are associated with a nongastric location, clinicopathologically aggressive features, and hyposensitivity to imatinib. GISTs with *KIT* exon 17 mutations are rare and some of them (e.g., D816V) are resistant to imatinib. *PDGFRA* mutations (present in 10 % of primary GISTs) are common in tumors of the stomach and have epithelioid features as well as indolent behaviors. The commonest mutation of *PDGFRA*, D842V, is associated with resistance to imatinib, sunitinib, and regorafenib. Approximately 10 % of GISTs are negative for *KIT* and *PDGFRA* mutations; these are referred to as wild-type GISTs. Wild-type GISTs are heterogeneous in genotype and may include mutations in *HRAS*, *NRAS*, *BRAF*, *NF1* or the SDH complex (Table 1). Wild-type GISTs may be considered insensitive to imatinib. A mutation analysis may add prognostic information for GIST patients, especially for some specific subtypes, and genotyping can provide critical biomarkers to predict the activity of TKIs. Pathology reports may include, at least, the pathological diagnosis of GIST, tumor origin, presence of preoperative or intraoperative rupture, histological type, maximal size (cm), mitotic index (area of 5 mm^2^), surgical margin, immunohistochemical findings (KIT, DOG1, CD34, desmin, S100, Ki67), presence of pathological necrosis, risk stratification, and *KIT* and *PDGFRA* mutations.

**Table 1:** Mutations and clinicopathological features

Genes	Exon	Frequent mutations	Frequency	Characteristics and site	Imatinib sensitivity
*KIT*	All exons		80 %	All sites	
	8		Rare	Small bowel	Yes, intermediate
	9	Insertion of AY 502–503	5–10 %	Small bowel, colon, spindle, aggressive	
	11	Deletions, missense mutations, insertions	60–70 %	All sites	Yes
		Deletion of codon 557 or 558		Aggressive, poor prognosis	
		Internal tandem duplication		Benign features, clinically indolent, female, stomach	
	13	K642E	1 %	All sites	Yes
	17	D820Y, N822K, Y823D	1 %	All sites	No for D816V
*PDGFRA*	All exons		10 %	Epithelioid, clinically indolent	
	12	Missense mutations	1–2 %	All sites	Yes
	14	N659K	<1 %	Stomach, epithelioid	Yes
	18	D842V	10–5 %	Stomach, mesentery, omentum, epithelioid	No for D842V
Wild-type			10–15 %	All sites	Probably no
*BRAF*		V600E	Rare		
*SDHA*/*SDHB*/*SDHC*/*SDHD* mutations			~2 %	Carney–Stratakis syndrome^a^; stomach, multiple, immunohistochemically SDHB negative	
				Juvenile GIST; stomach, clinically indolent, multiple, immunohistochemically SDHB negative	
Loss of SDH expression				Carney triad^b^; stomach, clinically indolent, juvenile onset, immunohistochemically SDHB negative	
*HRAS*, *NRAS* mutation			<1 %		
*NF1* mutation			1–2 %	Small bowel, clinically indolent, multiple, spindle	

*SDH* succinate dehydrogenase, *SDHB* succinate dehydrogenase iron–sulfur subunit (subunit B)

^a^Carney–Stratakis syndrome: familial syndrome of multiple GIST and paragangliomas with autosomal dominant inheritance and germline mutation in the SDH complex

^b^Carney triad: coexistence of gastric gastrointestinal stromal tumor (GIST), pulmonary chondroma, and extra-adrenal paraganglioma in young women, postulated to be defect in expression of the SDH complex

There are several subsets of GISTs with features distinct from those of conventional *KIT*- or *PDGFRA*-mutated GISTs, including pediatric GIST, neurofibromatosis type 1 associated GIST (NF1-GIST), Carney–Stratakis syndrome, the Carney triad, and familial GISTs (Table 1):

1. Pediatric GISTs, which are predominantly found in the female stomach, are frequently associated with predominant epithelioid features, lymph node metastasis, and mutations in the SDH complex. These tumors are sometimes multicentric and/or multinodular, and typically progress slowly. *SDH*-mutated GISTs are thought to be insensitive to imatinib, but sunitinib may work to some extent [10, 18].
2. NF1–GISTs are marked by wild-type and multicentric tumors, are predominantly located in the small intestine, and are relatively indolent in terms of clinical and pathological features. NF1–GISTs are insensitive to imatinib.
3. Carney–Stratakis syndrome is caused by germline loss-of-function mutations in SDH genes, including subunits A, B, C, and D, and is characterized by a dyad of gastric GIST and paraganglioma.
4. The Carney triad is typically marked by gastric GISTs, paraganglioma, and pulmonary chondromas and may be accompanied by an epigenetic loss of SDH expression.
5. Familial GISTs with germline mutations in either the *KIT* gene or the *PDGFRA* gene present as autosomal dominant traits and are associated with the presence of multiple GISTs in the gastrointestinal tract that are found in relatively young individuals [19].

### Diagnostic imaging

**Fig. 2:**
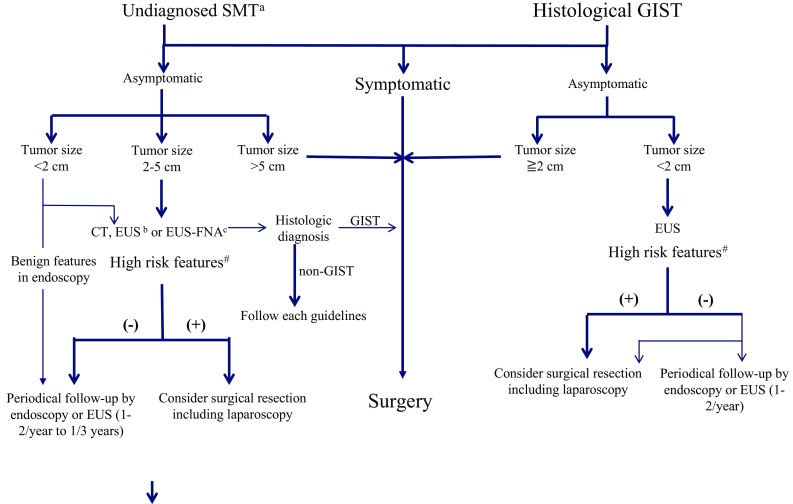
Diagnostic and therapeutic strategies for histologically undiagnosed gastric submucosal tumor (*SMT*) and histologically diagnosed gastric gastrointestinal stromal tumor (*GIST*). High-risk features include ulceration, irregular borders, internal heterogeneity, enlargement of regional lymph nodes, and an increase in size during follow-up. *CT* computed tomography, *EUS* endoscopic ultrasonography, *EUS-FNA* endoscopic-ultrasonography-guided fine-needle aspiration biopsy

Most GISTs are detected by endoscopy as a submucosal tumor (SMT), and the pathological diagnosis is often made after surgery. When small esophageal or gastric nodules (SMTs smaller than 2 cm) having no high-risk features are detected, they can usually be followed by periodic endoscopic ultrasonography (EUS) until the tumors increase in size or become symptomatic (Fig. 2), even if they are histologically GISTs [3, 4, 16]. Alternatively, the decision-making process can be shared with patients regarding whether to make a histological diagnosis—for example, by EUS-guided fine-needle aspiration (EUS-FNA) biopsy, or whether the patient should undergo further treatment. Although evidence to determine the optimal follow-up schedule is lacking, most guidelines recommend an initial short-term follow-up within 6 months by EUS (Fig. 2), followed by a more relaxed follow-up when there is no evidence of growth, high-risk features, or symptoms [3–5]. A recent retrospective study indicates that a relaxed follow-up did not worsen the prognosis of gastric GIST patients [12]. For rectal GISTs, however, the ESMO guidelines recommend surgical resection regardless of tumor size because the risk of rectal GIST is high and local control is critical. Although endoscopic removal of small GISTs has been reported, the safety and oncologic outcomes have not been established owing to the risks of positive margins, tumor spillage, and potential perforation. Therefore, endoscopic resection of SMTs is an investigational measure and should be performed only as part of a clinical trial in specialized centers [5, 6].

EUS-FNA biopsy may provide the most reliable histological diagnosis of SMTs before surgery. Its indications include histologically undiagnosed SMTs that may require medical or surgical treatment depending on their histological characteristics, such as neoadjuvant therapy for marginally resectable GISTs. EUS-FNA biopsy is not recommended for tumors for which it has already been planned that they will be resected by surgery, undoubtedly benign tumors, and small tumors (less than 2 cm) [20]. Because of the diagnostic limitations of endoscopy for GISTs/SMTs showing extrinsic growth, contrast-enhanced computed tomography (CT) and/or EUS is also recommended for GISTs/SMTs larger than 2 cm in an initial workup to evaluate whole images of tumors and high-risk features [5, 12, 16], unless they are obviously benign tumors. The Japanese GIST guidelines recommend that GISTs/SMTs larger than 5 cm, except for definitely benign tumors, should be resected by surgery and should be subjected to subsequent pathological examinations [5, 16].

### Prognostic factors and risk stratification

Independent prognostic factors for GIST include the mitotic index, tumor size, tumor location (gastric vs. nongastric), and tumor rupture [21]. Tumor rupture should be considered separately with regard to whether it occurred before or during surgery. Although the type of mutation(s) may add important prognostic information for risk assessment, the four factors mentioned above provide much more useful information in the prognostic stratification than the genotype does [22, 23].

Discrimination of benign GIST from malignant GIST by simply tumor diameter or mitotic index is not yet feasible; therefore, risk classification and nomograms have been introduced [23–26]. Of these, a risk-stratification procedure using tumor size and mitotic index, the National Institutes of Health (NIH) classification, is the method most frequently used in clinical trials because of the historical context [24], whereas the risk-classification method proposed by Miettinen and Lasota [25] that incorporates tumor size, number of mitoses, and tumor location is commonly used in daily clinical practice. The more recently proposed “modified NIH classification” is defined by four factors—number of mitoses, size, location, and rupture—and might offer advantages in the selection of patients who may require adjuvant therapy [23]. Nomograms can be used to estimate an individual’s risk of recurrence [26] and may be useful for individual decision-making with respect to adjuvant therapy. When tumor size and mitotic index are near the cut-off values, patients and physicians may discuss the information pertaining to estimated recurrence risk obtained from prognostic contour maps [23].

## Surgery for primary GIST

Surgery remains the only modality that can offer a permanent cure of GIST, and complete surgical resection avoiding tumor rupture and injuries to the pseudocapsule is the initial treatment for primary and localized GISTs when the risk of morbidity and death from surgery is acceptable. The aims of surgery include complete resection with macroscopic and microscopic negative margins and functional preservation by wedge resection, when applicable. The management of a positive microscopic margin after macroscopic complete resection is not well defined, and options may include re-excision, watchful waiting, and postoperative imatinib therapy. The information regarding the margin status and postoperative therapy should be shared with patients, and a multidisciplinary team should be involved in clinical decision-making. A recent retrospective analysis of clinical studies suggested that the margin status may have no significant prognostic effect in this era of targeted therapy [27]. Lymph node metastasis is very rare in GIST, and prophylactic dissection of lymph nodes is not necessary, except for the SDH-mutated GISTs [9], for which pickup dissection of swollen lymph nodes may be indicated.

Laparoscopic surgery may be successful for small gastric GISTs under the same oncological principles as for open surgery. In laparoscopy, direct handling of tumors with forceps is contraindicated, and a plastic bag should be used to minimize the risk of tumor seeding when the tumor samples are removed. Several retrospective cohort studies have suggested that laparoscopic resection is feasible and safe for gastric GISTs smaller than 5 cm and is less invasive than open surgery, with similar oncological outcomes [28]. The ESMO guidelines state that a laparoscopic approach is acceptable for small GISTs; however, it is not recommended for large tumors because of the risk of tumor rupture. Likewise, the NCCN guidelines state that laparoscopic resection is a reasonably safe and feasible procedure for patients with gastric GISTs 5 cm or smaller and that data on laparoscopic resection of GISTs other than gastric ones or ones larger than 5 cm are limited and laparoscopic surgery for these GISTs is not always recommended. The indications for laparoscopic surgery may depend on the anatomic site, developmental ways of tumors (e.g., intraluminal or extraluminal growth), and possibly on the level of experience of the multidisciplinary team. The indications for and role of laparoscopic or laparoscopic-assisted surgery have not been determined for GISTs larger than 5 cm or intestinal GISTs.

## Medical therapy for recurrent/metastatic GIST

### Imatinib mesylate

**Fig. 3:**
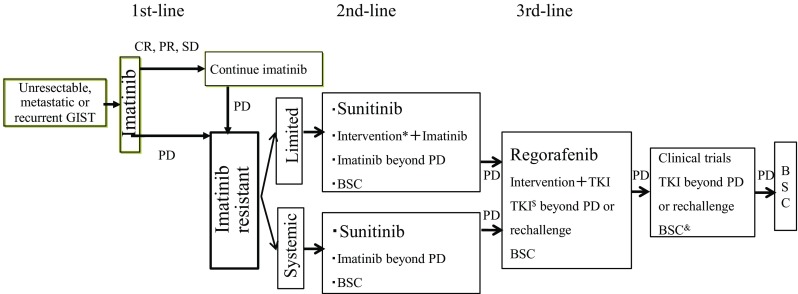
Treatment algorithm for unresectable, metastatic, or recurrent gastrointestinal stromal tumor (*GIST*). Interventions include surgical resection, radiofrequency ablation, and transcatheter arterial embolization for patients with limited progression. Tyrosine kinase inhibitors (*TKI*) include imatinib, sunitinib, and regorafenib, *BSC* best supportive care, *CR* complete response, *PD* progressive disease, *PR* partial response, *SD* stable disease

Imatinib mesylate is a first-line standard therapy for inoperable, metastatic, or recurrent GISTs (Fig. 3). The standard dosage is 400 mg/day. The NCCN and ESMO guidelines recommend a higher dosage for *KIT* exon 9-mutated GISTs because the higher dosage (800 mg/day) showed a longer progression-free survival (PFS) in such cases in a clinical trial [29]. However, treatment with more than 400 mg/day is not reimbursed in some countries, including Japan. The Japanese guidelines indicate that a higher dosage for exon 9-mutated GISTs is an optional approach. Previous reports found that a higher dosage of imatinib was associated with severer toxic effects than the standard dosage [31, 32], and individual optimization of imatinib therapy is mandatory. Interruption of imatinib treatment is accompanied by disease progression [33], so imatinib therapy should be continued indefinitely when tolerable, even after a complete response or macroscopic resection of residual tumors. It has been reported that half to two thirds of patients with metastatic/recurrent GISTs may show an objective response after imatinib treatment, and the median PFS is more than 2 years, with nearly 15 % of patients showing a durable response lasting more than 10 years [30, 31, 33, 34]. It may take several months to obtain a therapeutic effect in some cases, and the median time to response was 3 months [2]. More importantly, patients with stable disease lasting more than 6 months show oncological outcomes similar to those with an objective response [34], suggesting that careful monitoring of the tumor response is important in the early phases of treatment. However, 10–15 % of GIST patients show intolerance or resistance (primary resistance) to imatinib.

Biomarkers of the activity of imatinib may include the genotype and plasma levels of the drug [35, 36]. GISTs with *KIT* exon 11 mutations are most sensitive to imatinib, whereas those with *KIT* exon 9 mutations are less sensitive and may require a higher dosage (800 mg/day) to achieve longer PFS. GISTs with specific mutations, such as *PDGFRA* exon 18 (D842V) or *KIT* exon 17 (D816V) mutations, are resistant to imatinib [37]. Wild-type GISTs, which have no mutation in the *KIT* and *PDGFRA* genes, are also thought to be insensitive to imatinib. Thus, mutation testing is recommended when imatinib is being considered for treatment. Suboptimal plasma levels of imatinib might be associated with short PFS [38]. Assessment of the plasma drug level may be useful when there are unexpected toxic effects, suspected poor adherence, possible major drug–drug interactions, or unexpected early progressive disease under standard dosing.

Resistance to imatinib may include primary and secondary resistance: the former is associated with progressive disease within 6 months and the latter is associated with progressive disease after 6 months. The major causes of primary resistance are genotypes such as the D842V mutation, wild-type subtypes, and some *KIT* exon 9 mutations; those associated with secondary resistance are new mutations in two kinase domains that occur in the ATP-binding pocket or activation loop during imatinib therapy [39]. Secondary mutations are more frequently observed in GISTs with *KIT* exon 11 mutations than in those with *KIT* exon 9 mutations. Secondary mutations show clonal evolution, and newly acquired mutations are highly heterogeneous with regard to the metastatic sites in a patient, and sometimes even within a particular tumor nodule, although the primary mutation is the same throughout all lesions.

### Sunitinib malate

When GIST patients have progressive disease under imatinib treatment or are unable to tolerate imatinib because of adverse events, sunitinib malate (Sutent; Pfizer, New York, NY, USA) is recommended [40]. For some imatinib-resistant GISTs showing focal progression, resistant lesions may be treated by surgical resection, radiofrequency ablation, or transcatheter arterial embolization (Fig. 3). Surgery for limited progression has been shown to lead to a PFS of 6–12 months in retrospective studies [41–43], suggesting that these approaches for limited progression with continuing imatinib treatment may be useful. These treatments, however, are not well established and should be performed as investigational therapy by a multidisciplinary sarcoma team. The other option is dosage escalation of imatinib (to 800 mg/day), which may result in a prolongation of the median time-to-progression by 3 months [44].

Sunitinib is a multitarget inhibitor that inhibits KIT, platelet-derived growth factor receptors, vascular endothelial growth factor receptors 1–3, colony stimulating factor 1 receptor, and RET. The drug (50 mg/day) was initially approved for imatinib-resistant disease or intolerant patients with a 4-week-on/2-week-off schedule; the continuous use of 37.5 mg/day was later approved in the USA and EU but has not been approved in Japan. The dose and schedule may be individualized depending on patient response and adverse events. The reported response rate was nearly 10 %, and the clinical benefit rate was approximately 50 %, with a median PFS of 8 months, which was more than four times longer than that for the placebo [40]. The commonest treatment-related adverse events show a profile different from that for imatinib and are generally severer than those for imatinib. The activity of sunitinib is related to the primary and secondary mutations. With regard to the primary mutations, patients with *KIT* exon 9-mutated GISTs and wild-type GISTs receive more benefit from sunitinib treatment than do those with *KIT* exon 11 mutations. Regarding secondary mutations, patients who had GISTs with secondary mutations in the ATP-binding domain showed better responses and a better prognosis under sunitinib treatment than did those with mutations in the activation loop domain [45].

### Regorafenib

Regorafenib, another multitarget inhibitor, inhibits KIT, platelet-derived growth factor receptors, vascular endothelial growth factor receptors 1-3, fibroblast growth factor receptor, RET and BRAF. Regorafenib (160 mg/day) was initially used for imatinib- and sunitinib-resistant GIST with a 3-week-on/1-week-off regimen and resulted in a response rate of 4.5 %, a clinical benefit rate of nearly 50 %, and a median PFS of 5 months [46]. Therefore, the third-line therapy for GISTs progressing under sunitinib treatment is regorafenib (Fig. 3). Another option is rechallenge with imatinib after progression under sunitinib treatment, which showed a twofold increase in PFS (1.8 months) compared with placebo [47]. Surgical treatment for focally progressing lesions under sunitinib treatment may work for exceptional cases [48].

### Multidisciplinary management

After complete resection, nearly 60 % of GIST patients are cured by surgery alone; however, the other 40 % have relapses and require additional targeted therapy [21]. To improve the prognosis of patients with a substantial risk of recurrence, all the guidelines recommend adjuvant therapy with imatinib for 3 years, which improves not only relapse-free survival but also the overall survival of high-risk patients or those with ruptured GISTs [49]. Spontaneously ruptured GISTs, which may lead to spillage of tumor cells in the abdominal cavity, are thought to be accompanied by a very high risk of peritoneal recurrence. The optimal duration of adjuvant therapy for these patients is currently unknown. The use of adjuvant therapy is not recommended for very low risk or low-risk GIST, but there is no consensus for intermediate-risk GIST. The expected duration of treatment and the risks and benefits of treatment should be shared with patients. Mutation testing is critical in decision-making regarding the use of adjuvant therapy. None of the guidelines recommend adjuvant imatinib therapy for *PDGFRA* D842V mutations. In addition, wild-type GISTs are not considered candidates for adjuvant treatment; however, evidence is lacking for this contraindication. For patients with exon 9 mutations, a higher dosage of imatinib (800 mg/day) may be considered for adjuvant therapy, but there is a lack of evidence and some regulatory limitations, especially in Japan.

When patients are expected to have considerable morbidity and loss of organ functions after initial surgery, and when safety of surgery and organ-function sparing are anticipated after cytoreduction, preoperative imatinib therapy is recommended for very large and marginally resectable GISTs. Preoperative imatinib therapy does not increase the risk of complications of surgery, and the treatment has been shown to be feasible and safe; however, its long-term prognostic effects are still unclear [50]. Approximately 6 months of preoperative therapy may be considered if imatinib is active, but there is no established evidence regarding the length of preoperative therapy. In addition, these patients may require adjuvant therapy for 3 years to improve their prognosis. If the tumors progress during preoperative therapy, surgery is recommended after imatinib treatment has been promptly stopped. Thus, an initial evaluation of the activity of imatinib— for instance, within 1month of treatment starting—is important.

## Monitoring and follow-up

### Imaging follow-up after treatment

All the clinical practice guidelines contain a follow-up policy based on expert consensus. However, the recommendations for follow-up differ among the NCCN, ESMO, and Japanese and other countries’ guidelines in some aspects. A small tumor burden is associated with a better prognosis for TKI therapy. The objectives of follow-up after complete surgery may be early detection and treatment of relapses [51]. Abdominal and pelvic CT with contrast medium is sufficient for conventional follow-up of GIST patients because metastases outside the abdomen are very uncommon. Magnetic resonance imaging is an alternative to CT, especially in young patients. The frequency of imaging should be adjusted according to the risk of recurrence and the timing and conditions of treatment [52]. NIH, Armed Forces Institute of Pathology, or modified NIH risk stratification should be performed when the risk of recurrence is estimated after surgery. Annual abdominal CT for 5 years after surgery is thought to suffice for most patients with a less than intermediate risk of recurrence [51]. The trade-offs between early detection of recurrence and cumulative radiation exposure from repeated CT should be considered for patients with very low risk and low-risk GISTs. Recurrence risk after surgery is highest during the initial few years after surgery and decreases gradually thereafter, and the patients being treated with adjuvant imatinib therapy are at low risk when imatinib is active. However, their risk of recurrent GIST increases substantially during the first few years after the discontinuation of adjuvant imatinib therapy [49]. Thus, for high-risk GIST patients treated with adjuvant therapy, follow-up imaging may be done at 6-month intervals during the treatment, every 3–4 months during the first 2 years after adjuvant therapy has been stopped, and then once every 6–12 months for up to 10 years after surgery [51]. When patients have no adjuvant therapy, an interval of 3–4 months between imaging studies may be recommended during the initial few years after surgery.

### Imaging in response evaluation

Evaluating the response is occasionally challenging, especially in the early and late phases of TKI therapy. The effects of imatinib may appear as tumor shrinkage and a decrease in CT density in the presence of contrast enhancement [53]. In principle, it is advisable that the response to TKIs be evaluated according to the Response Evaluation Criteria in Solid Tumors [54]. In some cases, however, the tumor size may increase in spite of a decrease in tumor density and substantial symptomatic improvement after imatinib therapy; this increase is associated with a subsequent gradual decrease in size and eventually durable stabilization of the disease. Thus, modified CT response evaluation criteria could be applicable for imatinib therapy [3, 53]. This is not always true for sunitinib and regorafenib [55]. Disease progression may present as new lesions, a significant increase (more than 10 %) in the size of existing tumors, or the appearance of small intratumoral nodules with contrast enhancement even if there is no change in the overall tumor size [56]. Although conclusive data are lacking regarding the optimal monitoring interval during imatinib therapy, follow-up with CT every 3–6 months seems reasonable. When progression is suspected, the imaging frequency should be increased, and magnetic resonance imaging or contrast-enhanced ultrasonography may be considered as an alternative evaluation measure. Both 2-deoxy-2-[^18^F]fluoro-d-glucose positron emission tomography and positron emission tomography–CT have proven to be highly sensitive in the early assessment of the tumor response and are thought to be useful in cases with confusing responses in CT or in the early prediction of the response (e.g., preoperative treatment). However, a small proportion of GISTs (10–20 %) have no [18F]fluorodeoxyglucose uptake, and this modality is not always reimbursed in response evaluation, especially in Japan.

## Comments

The Japanese clinical practice guidelines for GIST were updated in 2014, and version 3.0 has been published in Japanese. This review is based on the ESMO guidelines, the new Japanese guidelines, and discussions with Asian experts. An official report by these experts will be published soon.

Toshirou Nishida, Seiichi Hirota, and Yuko Kitagawa are panel members for the Japanese clinical practice guidelines for GIST, Jean-Yves Blay is a panel member for the ESMO clinical practice guidelines for GIST, and Yoon-Koo Kang is a panel member for the clinical practice guidelines for GIST in Korea.

## Abbreviations

CT: Computed tomography
DOG1: Discovered on gastrointestinal stromal tumor 1
ESMO: European Society for Medical Oncology
EUS: Endoscopic ultrasonography
EUS-FNA: Endoscopic-ultrasonography-guided fine-needle aspiration
GIST: Gastrointestinal stromal tumor
NCCN: National Comprehensive Cancer Network
NF1-GIST: Neurofibromatosis type 1 associated gastrointestinal stromal tumor
NIH: National Institutes of Health
PFS: Progression-free survival
SDH: Succinate dehydrogenase
SDHB: Succinate dehydrogenase iron–sulfur subunit (subunit B)
SMT: Submucosal tumor
TKI: Tyrosine kinase inhibitor
